# Detection of Viable and Total Bacterial Community in the Pit Mud of Chinese Strong-Flavor Liquor Using Propidium Monoazide Combined With Quantitative PCR and 16S rRNA Gene Sequencing

**DOI:** 10.3389/fmicb.2020.00896

**Published:** 2020-05-26

**Authors:** Guangxun Tan, Rui Zhou, Wenqian Zhang, Yuanliang Hu, Zhiyong Ruan, Jing Li, Changyi Zhang, Dengjin Shen, Nan Peng, Yunxiang Liang, Shumiao Zhao

**Affiliations:** ^1^State Key Laboratory of Agricultural Microbiology, College of Life Science and Technology, Huazhong Agricultural University, Wuhan, China; ^2^Zhijiang Liquor Industry Co., Ltd., Zhijiang, China; ^3^Department of Biology, University of Copenhagen, Copenhagen, Denmark; ^4^Hubei Key Laboratory of Edible Wild Plants Conservation and Utilization, College of Life Sciences, Hubei Normal University, Huangshi, China; ^5^Key Laboratory of Microbial Resources (Ministry of Agriculture, China), Institute of Agricultural Resources and Regional Planning, Chinese Academy of Agricultural Sciences, Beijing, China; ^6^Carl R. Woese Institute for Genomic Biology, University of Illinois at Urbana-Champaign, Champaign, IL, United States

**Keywords:** 16S rRNA gene sequencing, Chinese strong-flavor liquor, pit mud, propidium monoazide (PMA), viable microbe detection

## Abstract

Microbiota in the pit mud (PM) plays a crucial role in the production of Chinese strong-flavor liquor (CSFL), the most popular distilled liquor in China. However, previous studies used total microbes, instead of viable ones, for the characterization of the microbial community in this environment. In this study, we used propidium monoazide (PMA) combined with quantitative polymerase chain reaction (qPCR) and 16S rRNA gene sequencing to verify the effect of non-viablee bacteria on the characterization of PM bacteria. After PMA concentration optimization, 50 μM PMA was chosen to pretreat 5 and 20 years PMs. The qPCR results showed that there were 50.78 and 71.84% of non-viable bacteria in the 5-year PM and 20-year PM, respectively. Both copy numbers of total bacteria and viable bacteria were significantly higher in 20-year PM than those in 5-year PM. Nevertheless, in terms of bacterial diversity and composition analyses at the operational taxonomic unit (OTU), phylum, class, and genus levels, 16S rRNA gene sequencing results displayed no significant differences between total bacteria and viable bacteria in both PM types. In conclusion, it is necessary for non-viable bacteria to be considered in determining absolute biomass of bacteria in PM, but not necessary in the analysis of diversity and composition of PM bacteria. To the best of our knowledge, our study is the first attempt to analyze viable bacteria in the PM of CSFL and provides useful information on how to accurately characterize a microbial community in a PM environment.

## Introduction

Traditional isolation and culture technology to study structure and diversity of microbial community have several limitations, as most microorganisms are not yet culturable (Rondon et al., 2000). Instead, DNA-based molecular technologies, such as amplification sequencing and metagenomic sequencing, have been widely applied (Carini et al., 2017). However, these methods have ignored DNA from non-viable microbes in the total DNA extraction. Non-viable microbes are defined as dead microbial cells with damaged membranes. As a result, the extracted total DNA actually comes from both viable microbes (i.e., live cells with intact membranes) and non-viable microbes. It is necessary to investigate the effect of non-viable bacteria on the analysis of the microbial community by DNA-based molecular technologies. Non-viable microbes were reported to affect the estimation of microbial community diversity in meconium (Stinson et al., 2019), clinical feces (Young et al., 2017), topsoil (Carini et al., 2017), rice wine (Lv et al., 2016), and cheese (Erkus et al., 2016), but not that in the samples of groundwater (Lopez-Fernandez et al., 2018) or soil battery (Gustave et al., 2019).

Chinese strong-flavor liquor (CSFL) is the most popular type of Chinese liquor, one of the six famous distilled liquors in the world (Luo et al., 2014a). The organoleptic properties of CSFL include fragrant flavor, soft mouthfeel, and long-lasting aftertaste (Zheng and Han, 2016). CSFL is produced from grains (such as sorghum, wheat, and rice) in cellars with a special solid fermentation technique followed by distillation (Hu et al., 2015). Jiuqu composed of fungi and bacteria is used as the starter for liquor fermentation (Hu et al., 2015). The cellar is a rectangular underground pit (2 × 3 × 2 m) covered with pit mud (PM) on the four walls and the base, and PM is a fermentation clay rich in microorganisms (Zheng et al., 2013; Tao et al., 2017). In a closed environment with relatively stable temperature (25–32°C), moisture (40–45°C), and pH (3.0–5.0), the microorganisms in Daqu and PM produce a variety of flavor substances, such as acetic acid, fatty acid, and ester, and play vital roles in liquor brewing (Luo et al., 2014b; Tao et al., 2017). In particular, ethyl caproate, produced from esterification of caproic acid and ethanol, is considered to be the key flavor substance enhancing CSFL quality (Tao et al., 2014). The cellar is usually in use for decades. During this period, the PM microorganisms maintain contact with fermentation substrates, from which PM microorganisms obtain stable nutrition for their long-term growth, reproduction, and metabolism before death (Zhao et al., 2017; Li et al., 2018).

The microbial structure of PM was studied frequently by sequencing methods focusing on total microbes in this environment (Xu et al., 2017; Zou et al., 2018a, b). One previous study on natural soils reported that non-viable DNA might obscure the subtle spatiotemporal patterns or treatment effects of diverse soil conditions (Carini et al., 2017). Given that non-viable microbes may be generated during CSFL production, it is necessary to evaluate the influence of non-viable microbes on the accurate characterization of the PM microbial community. Propidium monoazide (PMA), as a kind of DNA molecular dye, can enter non-viable microbes and interact with their DNA to inhibit DNA amplification (Lv et al., 2016). But PMA cannot enter live microbe cells and thus cannot affect their DNA molecules (Lv et al., 2016). By integrating PMA into conventional quantitative polymerase chain reaction (qPCR) and 16S rRNA gene high-throughput sequencing, we were able to remove non-viable bacteria from PM samples and then compare the differences in copy number, community diversity, and composition between the viable bacteria and total bacteria. This study will be conductive to accurate characterization of the microbial community in the PM of CSFL, and it will expand our knowledge of environmental microbiology.

## Materials and Methods

### PM Sampling

The PM samples were collected in May 2018 from Zhijiang Liquor Winery located in Yichang City, Hubei Province, China. Pit age is a key factor determining microbial structure (Ding et al., 2014). It was reported that prokaryotic diversity increased significantly before pit age of 25 years, and became stable thereafter (Tao et al., 2014). To study the influence of non-viable microbes on PMs with different ages, four 5-year and four 20-year fermentation pits continuously used for production were randomly selected. According to the sampling strategy described previously (Ding et al., 2015), each PM sample was a mixture of the samples from seven loci at the walls and the bottoms of a pit. In total, four copies of mixture samples from 25-year PM and four copies from 5-year PM were collected, respectively. Then, chemical properties of each PM sample were measured by Chinese standard protocols, including LY/T 1228-2015 for total nitrogen, LY/T 1232-2015 for phosphorus, LY/T1234-2015 for potassium, NY/T 1848-2010 for ammonia nitrogen, GB/T 11957-2001 for humic acid, GB7857-1987 for organic matter, and DB12/T 512-2014 for nitrate nitrogen. The pH was determined in a 1:5 suspension (2 g of pit mud added with 10 mL of deionized water). Afterward, each PM sample was homogenized and phosphate-buffered saline (PBS) (pH 7.4) was added to form a 1% (w/v) PM suspension that was then transferred to six 1.5-mL centrifuge tubes (1 mL of 1% PM suspension/tube) for further experiments.

### Positive Control Preparation and PMA Treatment

The positive control composed of non-viable *Escherichia coli* and PM suspension was designed to evaluate the PMA efficiency in each batch of PMA treatment. Non-viable *E. coli* was obtained by heating *E. coli* at 95°C for 30 min. Then 400 μL of the heated *E. coli* was centrifuged at 10,000 rpm for 5 min. After supernatant was removed, 1 mL of a 1% 20-year PM suspension was added to the precipitated *E. coli* to form a positive control. Considering the possibility of more non-viable bacteria in older PM than in younger PM, we used only 20-year PM to prepare positive control.

In each batch, 5-year PM, 20-year PM, and positive control (three copies) were treated with PMA according to the manufacturer’s instructions (GE-V001, GenEasy Inc., China). Briefly stated, 0, 10, 17.5, and 25 μL of 2 mM PMA respectively was added to 1 mL of 1% (w/v) PM suspension to obtain different PMA final concentrations of 0, 20, 35, and 50 μM. Meanwhile, 0 and 25 μL of 2 mM PMA was added to the positive control, respectively. Afterward, PMA-treated and untreated PM suspensions were mixed and incubated in the dark at 20°C for 5 min by using a photoreaction machine (GE-V004, GenEasy Inc., China). Subsequently, PM suspensions were exposed to light-emitting diode (LED) light by using the photoreaction machine for 5 min. This light exposure neutralized the DNA of non-viable cells, making it possible to detect DNA only from viable cells. Finally, PM suspensions were frozen at −20°C for DNA extraction.

### DNA Extraction

Total DNA was extracted from PM suspension using Fast DNA^®^ SPIN Kit for Soil (MP Biomedicals, Santa Ana, CA, United States) following the manufacturer’s instructions, and the extracted DNA was stored at −20°C before further use. DNA concentration was measured by NanoDrop 2000 UV-vis spectrophotometer (Thermo Fisher Scientific, Wilmington, DE, United States), and DNA quality was evaluated through 1% agarose gel electrophoresis.

### Quantitative PCR

For PCR amplification of bacteria, primers (515F and 806R) targeting the V4 region of 16S rRNA gene were used (Carini et al., 2017).

qPCR was used to estimate 16S rRNA gene copy number on the ABI StepOne Plus qPCR instrument (Applied Biosystems, Foster City, CA, United States). The 20 μL qPCR mixture solution consisted of 10 μL of UNICON^®^ qPCR SYBR^®^ Green Master Mix (Yeasen, Shanghai, China), 0.5 μL of forward primer, 0.5 μL of reverse primer, 2 μL of DNA template, and 7 μL of distilled water. The amplification procedure included an initial denaturation at 95°C for 5 min, followed by 40 cycles of denaturation at 95°C for 5 s, annealing at 56°C for 20 s, and extension at 72°C for 20 s. Standard curves of total bacteria were developed from PCR products of *E. coli* (Supplementary Figure S1). The amplification efficiency was 95.0%. All reactions were performed in triplicate. The Mann–Whitney *U* test was used to evaluate the difference in the 16S rRNA gene copy number between PMA-treated and untreated PM.

### High-Throughput Sequencing and Data Analysis

The 16S rRNA V4 region was amplified using forward primer 515F and reverse primer 806R. The PCR amplification was conducted as follows: initial denaturation at 94°C for 3 min, 25 cycles of denaturation at 94°C for 5 s, annealing at 57°C for 90 s, chain elongation at 72°C for 10 s, and final extension at 72°C for 5 min. The PCR products were sequenced on the Illumina MiSeq platform to generate 2 × 300 bp paired-end reads.

In the data analysis, assembly was performed with Pandaseq (Masella et al., 2012). The operational taxonomic unit (OTU) was clustered by Usearch (version 7.1)^1^ with a sequence identity of 97%. The OTU was normalized by rarefaction to the same number of reads (33,573 reads, the lowest read number of samples) in each sample; then the rarefied OTU was used for all the following analyses. One representative sequence of each OTU was selected by QIIME. The taxonomic identification of OTU was conducted by searching the representative sequence against the 16S databases (RDP)^2^ with the RDP method. The relative abundances at phylum, class, and genus levels were calculated according to the OTU annotations. The alpha diversity was determined with QIIME software. Principal coordinates analysis (PCoA) and differential test of Shannon index were carried out by in-house tools. STAMP software was used to test the differences in relative abundance of OTU, phylum, class, and genus between viable and total bacterial communities.

### Data Accessibility

16S rRNA gene sequencing data are available at the NCBI database with accession number PRJNA590694.

## Results

### PMA Concentration Optimization

**FIGURE 1:**
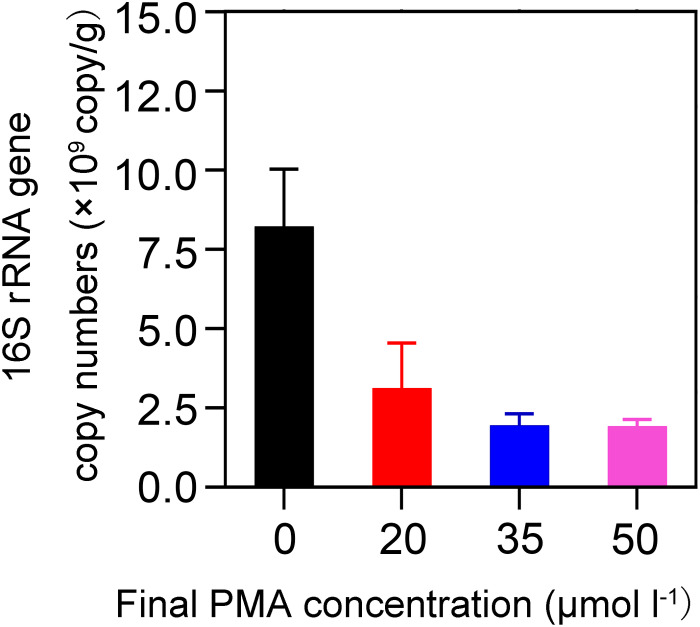
Effect of PMA concentration on detecting the abundance of 16S rRNA gene by qPCR. The quantities 0, 10, 17.5, and 25 μL of 2 mM PMA were added to 1 mL of 1% PM sample suspension, respectively, to obtain different PMA concentrations of 0, 20, 35, and 50 μM.

We investigated the effectiveness of removal of DNA from non-viable bacteria by PMA and evaluated the optimal PMA concentration. We used 20-year PM for the concentration optimization because we assumed that an optimal PMA concentration at which DNA could be removed from non-viable bacteria for 20-year PM was also applicable for 5-year PM. Our assumption was based on the following two findings: first, previous studies reported that the abundance of 16S rRNA gene increased with cellar age (Bei et al., 2014; Tao et al., 2014); second, we found 20-year PM exhibited significantly higher levels of pH, phosphorus, potassium, and organic matter than 5-year PM in this study (*t*-test, *P* < 0.05) (Supplementary Table S1). Based on the finding that the quantity of bacteria was positively correlated with the levels of pH, phosphorus, potassium, ammonia nitrogen, and humic acid (Wang et al., 2019), we speculated that 20-year PM should have more bacteria than 5-year PM. As shown in Figure 1, when compared with the PMs not treated with PMA (i.e., 0 μM), all the PMA-treated PMs had significantly reduced copy numbers of 16S rRNA gene, indicating that PMA could effectively inhibit DNA amplification of non-viable bacteria. However, we found that when PMA concentration increased from 0 to 35 μM, the copy number of 16S rRNA gene decreased dramatically, and that when PMA concentration increased from 35 to 50 μM, the copy number of 16S rRNA gene decreased slightly, which suggested that the concentration lower than 35 μM could not completely inhibit the DNA amplification of non-viable bacteria. Therefore, we chose 50 μM as the optimal PMA concentration for further analysis.

### Quantification of Total and Viable Bacteria

**FIGURE 2:**
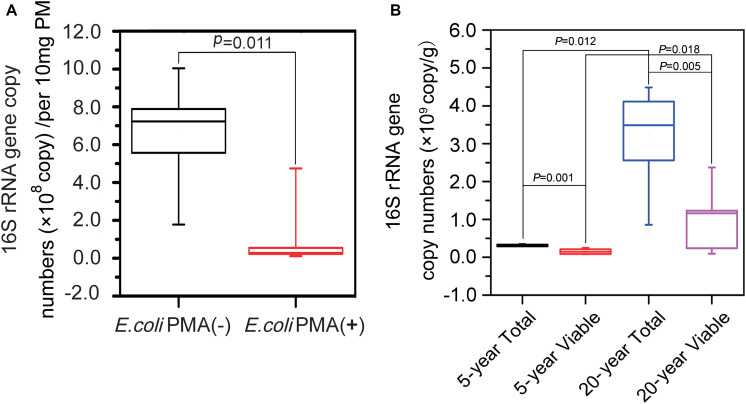
Abundance analysis of total bacteria and viable bacteria of the PMs by qPCR. **(A)** The positive control composed of dead E. coli and PM sample suspension was untreated [*E. coli* PMA (-)] and treated [*E. coli* PMA (+)] with PMA for quantifying total bacteria and viable bacteria, respectively. **(B)** 5-year PM and 20-year PM were untreated with PMA for quantifying total bacteria (5-year Total, 20-year Total) and treated with PMA for quantifying viable bacteria (5-year Viable, 20-year Viable). The Mann–Whitney *U*-test was used to compare the abundance of 16S rRNA genes by SPSS software.

PCR was used to amplify 16S rRNA genes in PM samples. In terms of PMA efficiency, we found that on average, 7.82 × 10^8^ 16S rRNA genes per 10 mg of PM in positive controls were suppressed from amplification after PMA treatment (Figure 2A). There were on average 3.10 × 10^6^ and 3.21 × 10^7^ copy numbers of 16S rRNA gene per 10 mg of PM in the total bacteria of 5-year PM and 20-year PM, respectively, which indicated that the abundance of non-viable bacteria in PM samples was far less than that in positive controls. The abundance of total bacteria was significantly higher than that of viable bacteria in both 5-year PM (Mann–Whitney *U-*test, *P* = 0.001) and 20-year PM (Mann–Whitney *U*-test, *P* = 0.005) (Figure 2B). These results demonstrated that PMA treatment at 50 μM concentration effectively removed non-viable bacteria in PM samples. In addition, we found that 20-year PM exhibited significantly higher abundance of bacteria including total bacteria, viable bacteria, and non-viable bacteria than 5-year PM. Our results were in consistent with our assumption of more bacteria in 20-year PM than in 5-year PM for designing PMA concentration optimization. Specifically, the total bacterium abundance in 20-year PM was 10.34 times as high as that in 5-year PM, while the viable bacterium abundance in 20-year PM was 6.37 times as high as that in 5-year PM (Figure 2B). We quantified non-viable bacteria by subtracting the copy number of viable bacteria from the copy number of total bacteria, and found that non-viable bacteria accounted for 50.78% ± 23.30% and 71.84% ± 18.12% of total bacteria in 5-year PM and in 20-year PM, respectively (Figure 2B).

### Diversity and Structural Analysis of Total and Viable Bacteria

High-throughput sequencing of 16S rRNA gene in PM samples generated a total of 851,633 reads with an average length of 276 bp. There were 33,573–71,127 reads in each sample (Supplementary Table S2). After rarefaction, 596 OTUs were retained from all the samples. A total of 22 phyla, 40 classes, and 283 genera were identified.

**FIGURE 3:**
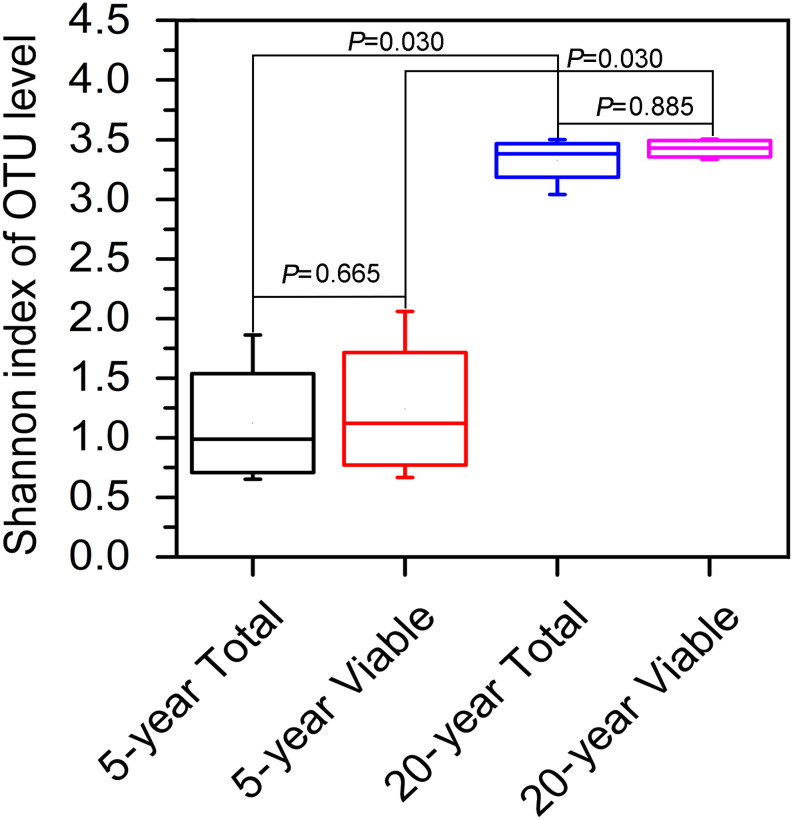
Shannon index based on OTUs in different PM samples. The 5-year PM and 20-year PM were treated with PMA to identify viable bacteria (5-year Viable, 20-year Viable), whereas they were not treated with PMA to identify total bacteria (5-year Total, 20-year Total). The non-parametric rank sum test was used to compare two groups of Shannon index.

In terms of Shannon index, no significant difference in bacterial diversity was observed between total bacteria and viable bacteria in 5-year PM (Wilcoxon rank sum test, *P* = 0.665) or 20-year PM (Wilcoxon rank sum test, *P* = 0.885), suggesting that non-viable bacteria had little effect on the estimation of bacteria diversity in PM samples (Figure 3). However, we found that both total bacteria diversity and viable bacteria diversity of 20-year PM were significantly higher than those of 5-year PM (Figure 3). Accordingly, the cellar age was an important factor affecting the bacterial diversity.

**FIGURE 4:**
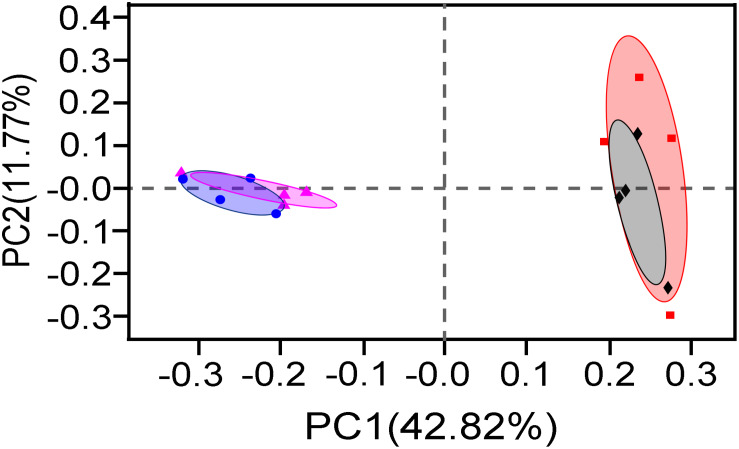
PCoA analysis of total bacteria and viable bacteria in different PM samples (◆) 5-year Total, (•) 20-year Total, (■) 5-year Viable, (▲) 20-year Viable.

Then, PCoA based on 16S rRNA gene OTUs was performed to examine the difference in bacteria communities (Figure 4). Two principal components (PC1 42.82% and PC2 11.77%) explained 54.59% total variations of bacterial community in the samples. The 20-year PM samples were obviously separated from 5-year PM ones along axis PC1, implying that two PM types had significantly different bacterial community structures. This result was then verified by ANOSIM analysis (5-year total bacteria vs. 20-year total bacteria, *R* = 0.9896, *P* = 0.034; 5-year viable bacteria vs. 20-year viable bacteria, *R* = 0.8854, *P* = 0.034). Meanwhile, PCoA result indicated that the community structure of total bacteria closely overlapped that of viable bacteria in either 20-year PM or 5-year PM. In consistent with this observation, ANOSIM analysis showed insignificant difference between total bacteria and viable bacteria in each PM type (5-year total vs. 5-year viable, *R* = 0.1875, *P* = 0.214. 20-year total vs. 20-year viable, *R* = 0.01, *P* = 0.445). Taken together, our results showed that microbial community structure of PM was affected by the cellar age, but not by non-viable bacteria.

### Composition Analysis of Total and Viable Bacteria

We analyzed the relative abundance of bacteria at different levels in order to evaluate the composition differences in subgroups of PM samples. The results of Wilcoxon rank sum test suggested that there were no significant differences in relative abundance between total bacteria and viable bacteria at OTU, phylum, class, or genus levels. The detailed relative abundance values of different bacteria taxa are listed in Supplementary Tables S3–S6 and Supplementary Figures S2–S5.

**FIGURE 5:**
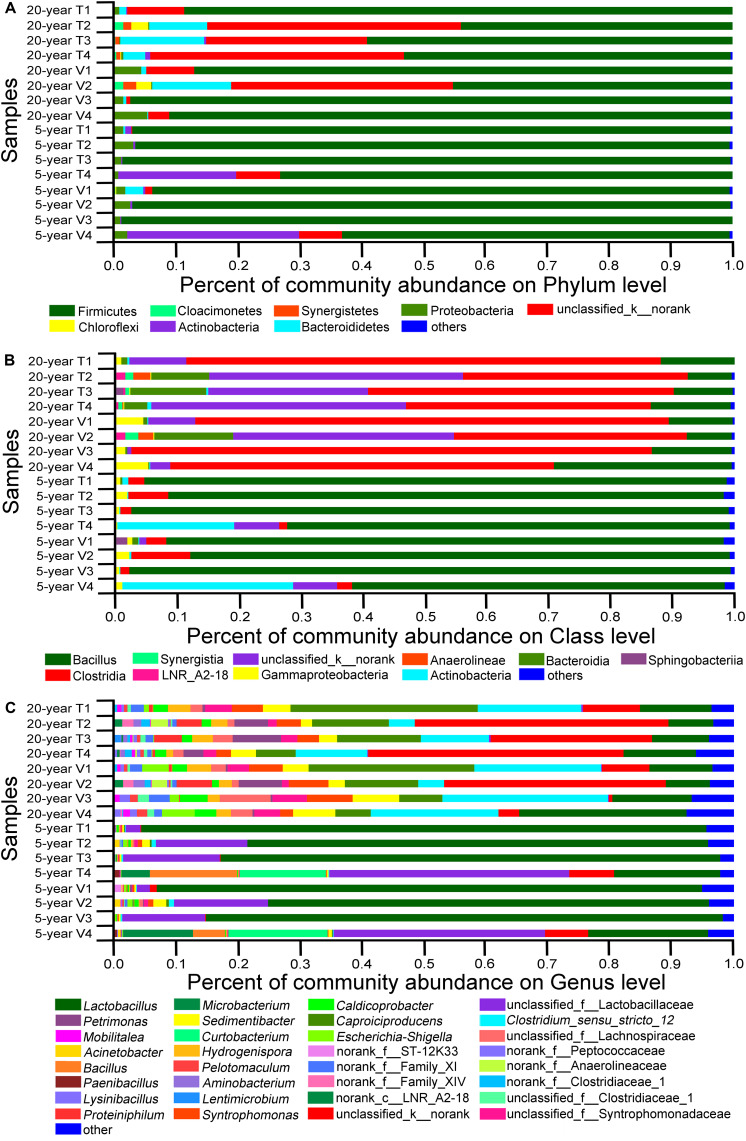
The relative abundances of viable bacteria at different levels of phylum **(A)**, class **(B)**, and genus **(C)**. Each PM type had four replicates. The 5-year PM and 20-year PM were treated with PMA for identifying viable bacteria. The prefixes of “k__,” “p__,” “c__,” “o__,” and “f__” indicate that OTUs were annotated only to the level of kingdom, phylum, class, order, or family.

Next, we studied the changes in the composition of viable bacterial communities in PMA-treated PM, since viable bacteria were considered to be vital for characterization of bacteria community. We defined dominant phyla as those detected in at least one PM sample and with the relative abundance higher than 1.0%. Dominant class and dominant genus were defined with the same criteria. In total, 8 dominant phyla, 10 dominant classes, and 32 dominant genera were determined across all the samples (Figure 5). By comparing the relative proportion of these dominant bacterial communities among PM samples (Figure 5), we found obvious difference in composition patterns of viable bacteria between 5-year PMs and 20-year PMs. At the phylum level (Supplementary Table S7), Firmicutes were the most abundant in 5-year PMs with average relative abundance higher than 80.0%, while Firmicutes and unclassified_kingdom_norank were the most abundant in 20-year PMs. And the relative abundances of Firmicutes in 20-year PMs were lower than those in 5-year PMs. At the class level (Supplementary Table S8), Bacilli were the most abundant in 5-year PMs; in contrast, Bacilli accounted for only a small proportion in 20-year PMs. And the most abundant classes in 20-year PM were Clostridia and unclassified norank. At the genus level (Supplementary Table S9), *Lactobacillus* and unclassified_family_Lactobacillaceae occupied 81.56% of total abundance in 5-year PM, but only 14.27% of total abundance in 20-year PM. In 20-year PM samples, *Lactobacillus*, *Clostridium*_*sensu_stricto*_12, unclassified_kingdom_norank, and *Caproiciproducens* accounted for 14.23, 17.97, 11.89, and 12.82% of relative abundance in viable bacteria, respectively. By contrast, the corresponding abundances of four genera in 5-year PM were 65.43, 0.40, 2.05, and 0.14% in viable bacteria, respectively.

## Discussion

Because DNA of dead bacteria can exist in soil for a long period, the effects of non-viable bacteria on the estimation of microbial community in the PM of CSFL should be considered. In this study, PMA treatment prior to the amplification of qPCR or 16S rRNA gene sequencing was performed to distinguish non-viable bacteria from total bacteria in PM samples. To ensure effective PMA treatment, PM suspension concentration was determined, PMA concentration was optimized, and a positive control group was set. First, a 1% PM suspension concentration was determined by referring to PMA instructions and a previous study (Carini et al., 2017). Second, PMA concentration was optimized, as soil samples with different textures generally required different PMA concentrations for suppressing DNA from non-viable microbes. The 50 and 40 μM concentrations were reported to be suitable for inhibiting DNA in fresh sludge (Tian et al., 2017) and in surface soils (Carini et al., 2017), respectively. Our study indicated that 35–50 μM PMA concentrations were useful for PM sample treatment, and that a 50 μM PMA concentration was selected as the optimal concentration for removing non-viable bacteria (Figure 1). Finally, a PM suspension to which was added 400 μL of dead *E. coli* was set as a positive control. Similar controls were reported in previous studies. Salmon DNA (Sketa DNA) was added into the wastewater samples for PMA treatment validation (Kibbee and Örmeci, 2017). Heat-shocked sludge was obtained by heating at 95°C for 15 min as a positive control in one previous study (Tian et al., 2017). Our results revealed that adding dead *E. coli* into 1% PM suspensions was useful for validating the effectiveness of PMA treatment (Figure 2A).

As non-viable bacteria shared a high proportion in the PM microbe, they should be removed to avoid the overestimation of communities. Comparison results of copy numbers of 16S rRNA genes across all samples indicated that the copy number of total bacteria was significantly larger than that of viable bacteria in every PM type. Non-viable bacteria accounted for 71.8% of total bacteria in 20-year PMs, which was much higher than the corresponding proportion (40.7%) reported in a previous study of soil (Carini et al., 2017). There are two possible reasons for the high proportion of non-viable bacteria in PM. First, a large number of small molecules (glucose, caproic acid, butyric acid, acetic acid, etc.) produced by fermentation may enter PM, providing sufficient nutrition for the large-scale reproduction of microbes (Tao et al., 2014; Zhao et al., 2017; Li et al., 2018). Afterward, these microbes died and formed a large number of non-viable cells. Second, non-viable bacteria could persist in the soil for several weeks, even several years before degradation (Levybooth et al., 2007; Nielsen et al., 2007). Actually, the long-term existence of non-viable bacteria was also reported in other environments and processes, such as surface soil (Carini et al., 2017), cheese ripening (Erkus et al., 2016), and rice wine brewing (Lv et al., 2016).

Non-viable bacteria had little effect on the estimation of the relative composition of PM bacteria. In this study, we found no significant difference in diversity between total bacteria and viable bacteria, although there existed large quantities of non-viable bacteria. Accordingly, the diversity estimated from total bacteria could be used to represent the diversity of viable bacteria. Our findings might be attributed to the facts that PM was covered by fermentation substrate for all year round with relatively stable temperature, humidity, acidity, and nutrient supply, and that PM was hardly affected by the outside environment. Therefore, there would be a dynamic balance between bacteria community and PM environment. In this case, birth and death of PM bacteria would become balanced and contribute to a stable community structure (Shang, 2010). Similar findings were reported in some previous studies (Ali, 2018; Lopez-Fernandez et al., 2018; Gustave et al., 2019). Gustave et al. (2019) proposed two possible explanations. First, some cells could be preserved in the soil and remain intact after death. Second, there might be an equilibrium between cell death and extracellular DNA degradation (Gustave et al., 2019). Lopez-Fernandez et al. (2018) attributed this to the fast degradation of non-viable cells under oligotrophic conditions. In contrary to this, relic DNA from environment such as soils was reported to cause large differences between total and viable bacteria communities (Carini et al., 2017).

Non-viable bacteria had no significant effect on the relative abundance of bacteria in almost all the PMs at four levels of OTU, phylum, class, and genus. In other words, the relative abundance findings in previous microbe studies without considering non-viable bacteria would still be valid. As mentioned earlier, we speculated that the insignificant effect might be due to the balance between birth and death of the bacteria in certain circumstances. In contrast, it was also reported that the relative abundances of some key microbial lineages in soil changed after the removal of non-viable cells, and that the change trend varied with species (Carini et al., 2017).

Non-viable bacteria had no impact on the quality of CSFL. It had been reported that non-viable microbes could affect the shelf life of milk products (Ouwehand and Salminen, 1998) and the immune regulation function of fermented milk formula (Ménard et al., 2006), because these microbes are the ingredients. In contrast, no PM bacteria is contained in the CSFL. CSFL is obtained by complex fermentation processes followed by distillation. As a result, only volatile aroma compounds and water in the fermented grains are volatilized to form liquor, whereas both viable and non-viable bacteria are retained in the PM. The non-viable bacteria might be converted into humus, some of which might be further exploited and decomposed by microorganisms (Li, 2000). Therefore, non-viable bacteria could not have any effects on the liquor quality, such as organoleptic quality, through distillation or active metabolism.

Unlike non-viable bacteria, viable bacteria had close associations with the liquor’s quality. To the best of our knowledge, our study makes a preliminary attempt to evaluate the composition of viable bacteria in PM. We found that the viable bacteria of 5-year PM was almost completely dominated by *Lactobacillus* (Supplementary Table S9). However, the viable bacteria of 20-year PM was dominated by four core genera, including *Caprocciproducens*, *Clostridium sensu* _*stricto*_12, *Lactobacillus*, and unclassified_kingdom_norank (Supplementary Table S9). Our results were in line with the previous report on total bacteria (Tao et al., 2014). It was previously reported that most bacteria of the genera *Lactobacillus* and *Lactococcus* could produce large amount of lactic acid (Zhang et al., 2005) and that the obtained lactic acid could form ethyl lactate (Liang et al., 2016). Previous studies reported that *Caproiciproducens* could produce caproic acid (Kim et al., 2015), and that *Clostridium sensu stricto*, as one kind of strictly anaerobic bacteria, was regarded as the true *Clostridium* (Chai et al., 2019). It could ferment cellulosic-based biomass and sugar to form butyric acid and eventually to produce ethyl caproate (Barker et al., 1945; Liu et al., 2019). Excessive levels of ethyl lactate might make the liquor harmony worse, while ethyl caproate is recognized as a key component favorably influencing the flavor of CSFL (Tao et al., 2014). Therefore, the young PM such as 5-year PM dominated by *Lactobacillus* could produce liquor with more ethyl lactate, and the old PM such as 20-year PM dominated by *Caproiciproducens* and *Clostridium sensu _stricto_*12 could produce liquor with more ethyl caproate than the young PM. These findings suggested the flavor of the CSFL produced in old PM was of a higher quality.

In summary, non-viable bacteria have a significant effect on the absolute quantification of the PM bacteria, while they have little effect on the bacterial diversity, structure, and relative abundance of PM. Therefore, we suggest the effect of non-viable bacteria should be taken into consideration when PM bacteria are absolutely quantified. In addition, 35–50 μM is the effective PMA concentration for inhibiting non-viable DNA in PM samples. This study provides an insight into the PM microbial community and increases the knowledge of environmental microbiology. Our findings can be applied as guidance for the production of Chinese strong-flavor liquor.

## Data Availability Statement

The 16S rRNA gene sequencing data are available at NCBI database with the accession number PRJNA590694.

## Author Contributions

GT and DS performed the experiments. GT, RZ, and WZ analyzed the data and wrote the manuscript. YH contributed to manuscript preparation and experimental design. GT, WZ, ZR, CZ, and NP contributed to manuscript revision. JL and YL provided expertise and insights relating to Chinese liquor microbiology. SZ contributed to experimental design, manuscript revision, and overall support of this study.

## Conflict of Interest

GT and JL were employed by Zhijiang Liquor Industry Co., Ltd. The remaining authors declare that the research was conducted in the absence of any commercial or financial relationships that could be construed as a potential conflict of interest.

## Footnotes

1 http://drive5.com/usearch/

2 http://rdp.cme.msu.edu/
