# Supplementary material for: Detection of Viable and Total Bacterial Community in the Pit Mud of Chinese Strong-Flavor Liquor Using Propidium Monoazide Combined With Quantitative PCR and 16S rRNA Gene Sequencing
Source: Front Microbiol. 2020 May 26;11:896. doi: 10.3389/fmicb.2020.00896 (PMC7264162; doi:10.3389/fmicb.2020.00896)
Supplement: Supplementary file 1 [file Data_Sheet_1.zip › 4Supplementary_Figures.docx]

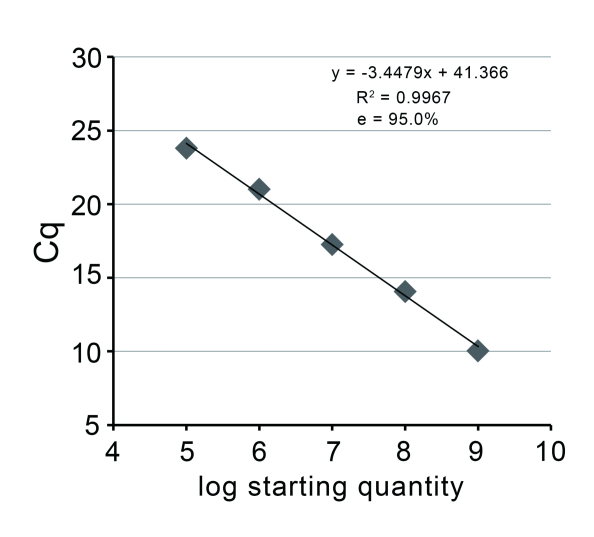


**Supplementary Figure 1. Standard curve of qPCR for total bacteria.**


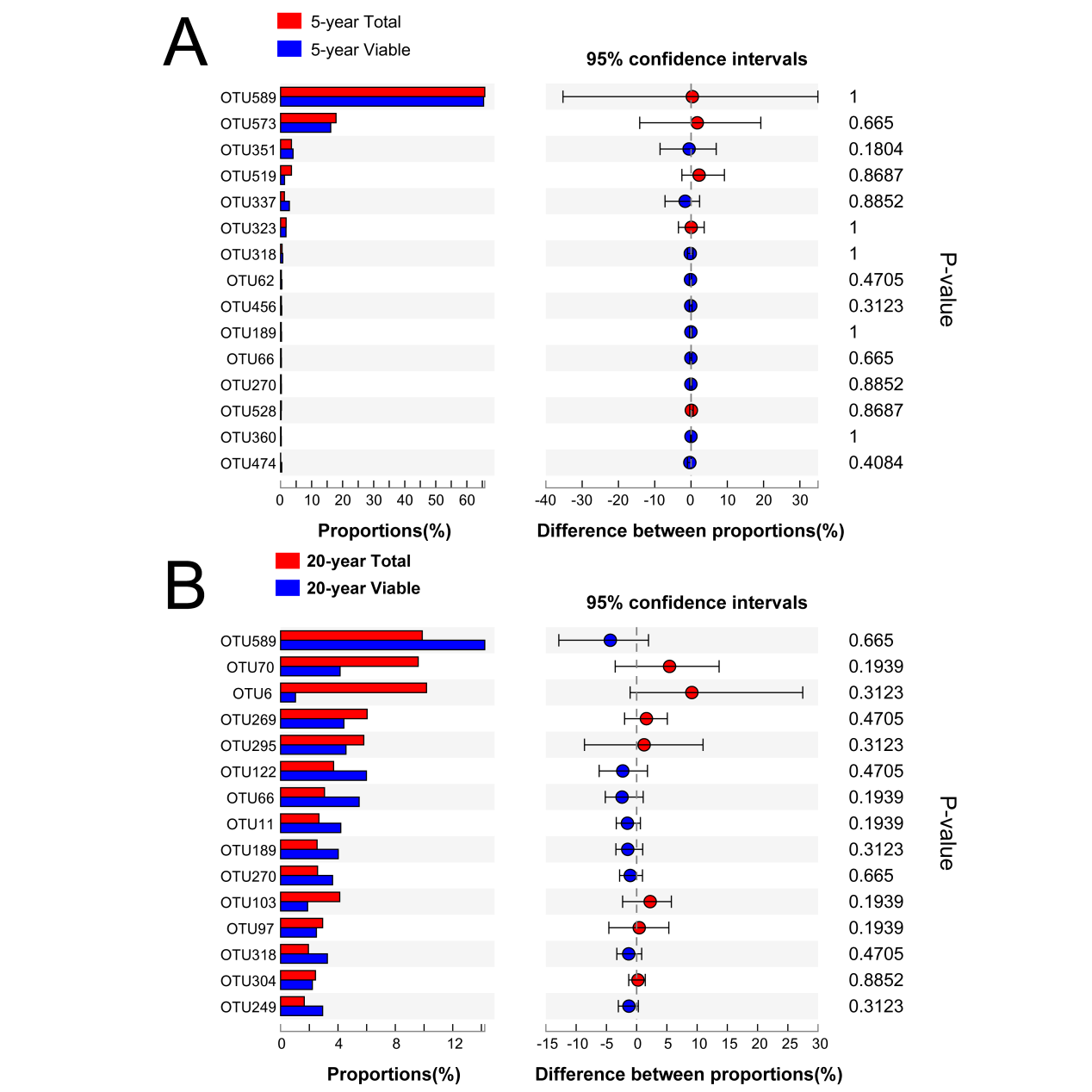


**Supplementary Figure 2. Statistically significant differences in the relative abundance of the top 15 OTU between total bacteria and viable bacteria in 5-year PM (A) and 20-year PM (B)**. 5-year PM and 20-year PM were treated with PMA for detecting viable bacteria (5-year Viable, 20-year Viable), and untreated with PMA for detecting total bacteria (5-year Total, 20-year Total).


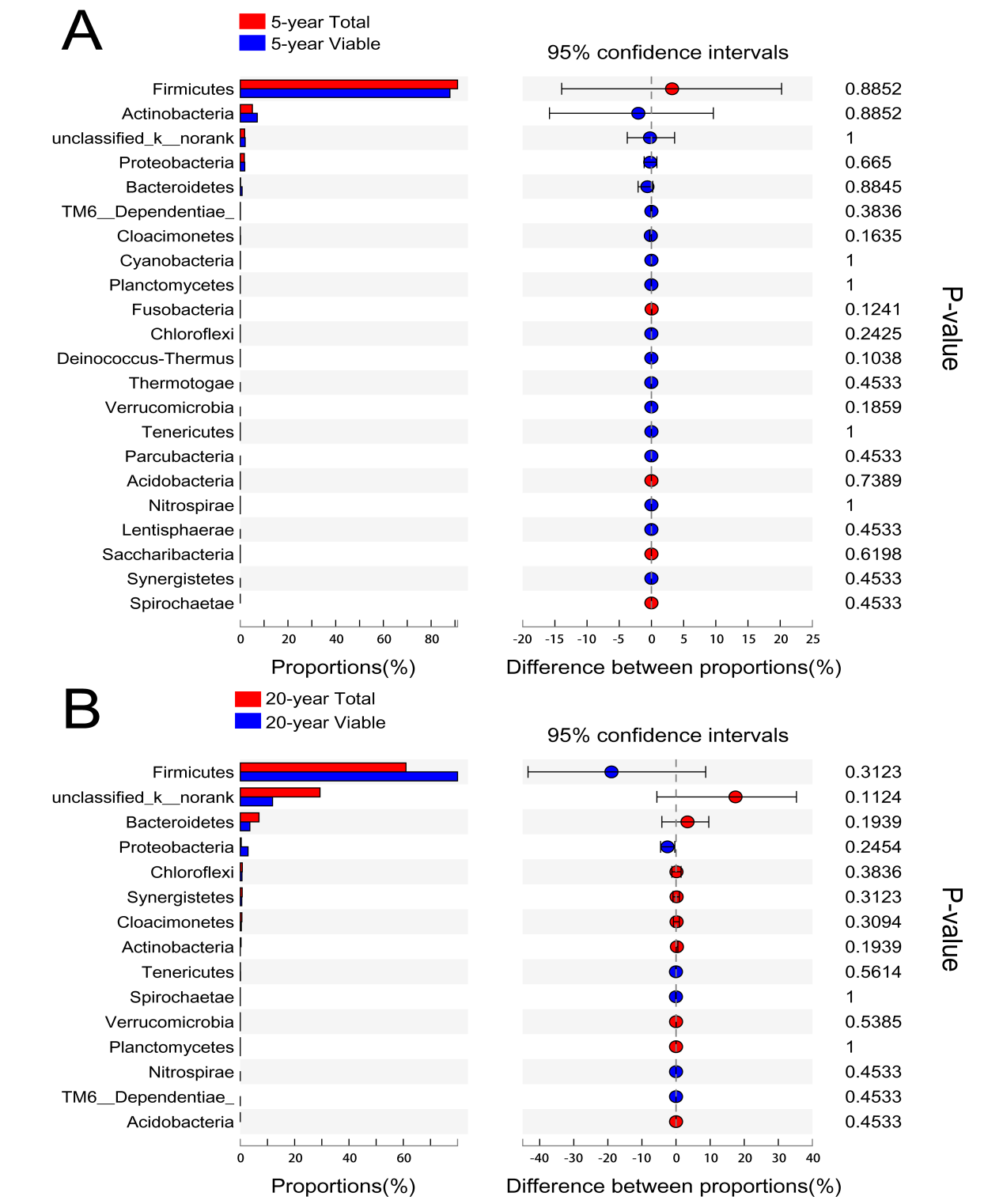


**Supplementary Figure 3. Statistically significant differences in the relative abundance of all the phyla between total bacteria and viable bacteria in 5-year PM (A) and 20-year PM (B)**. 5-year PM and 20-year PM were treated with PMA for detecting viable bacteria (5-year Viable, 20-year Viable), and untreated with PMA for detecting total bacteria (5-year Total, 20-year Total).


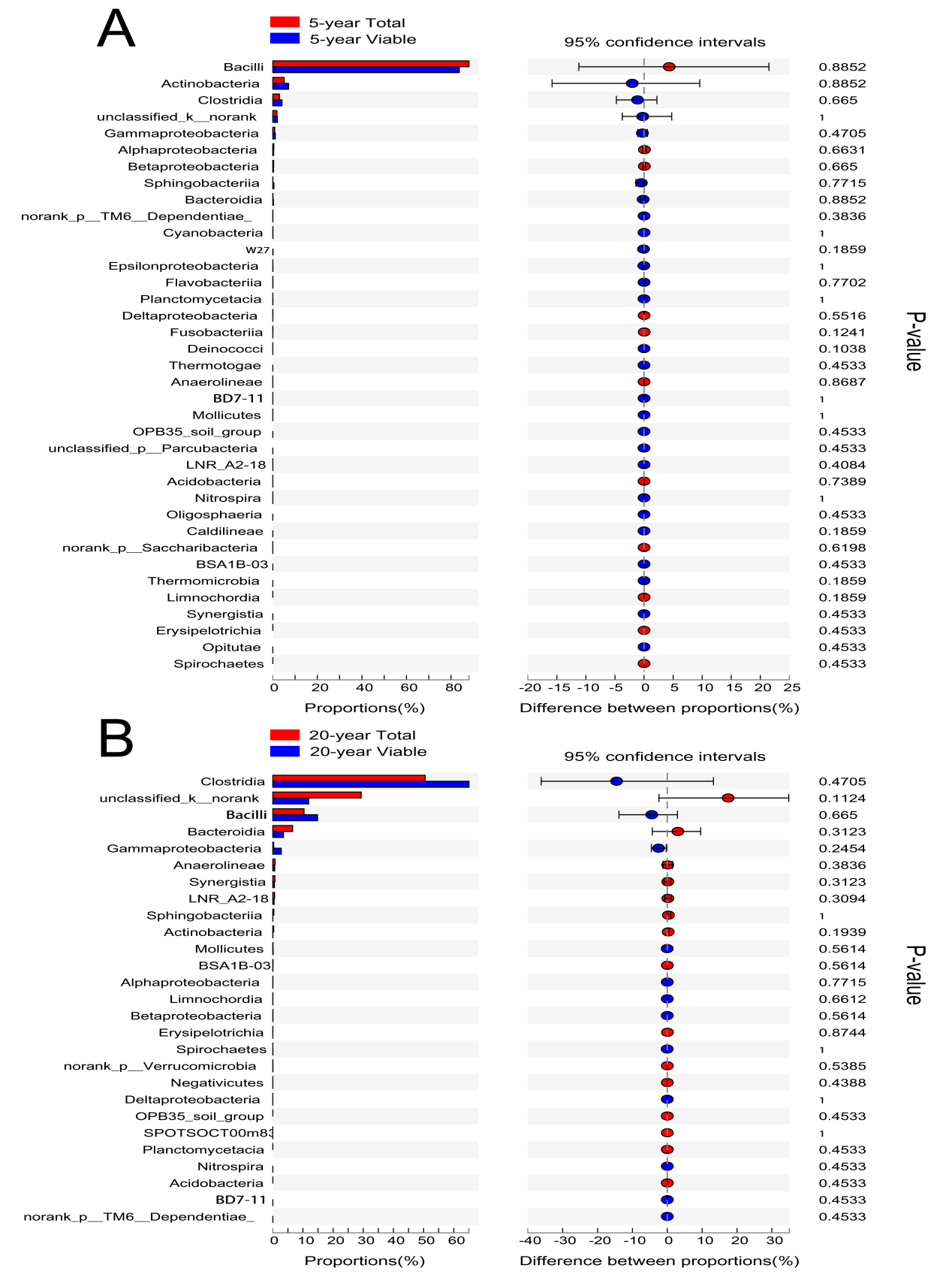


**Supplementary Figure 4. Statistically significant differences in the relative abundance of all the classes between total bacteria and viable bacteria in 5-year PM (A) and 20-year PM (B)**. 5-year PM and 20-year PM were treated with PMA for detecting viable bacteria (5-year Viable, 20-year Viable), and untreated with PMA for detecting total bacteria (5-year Total, 20-year Total).


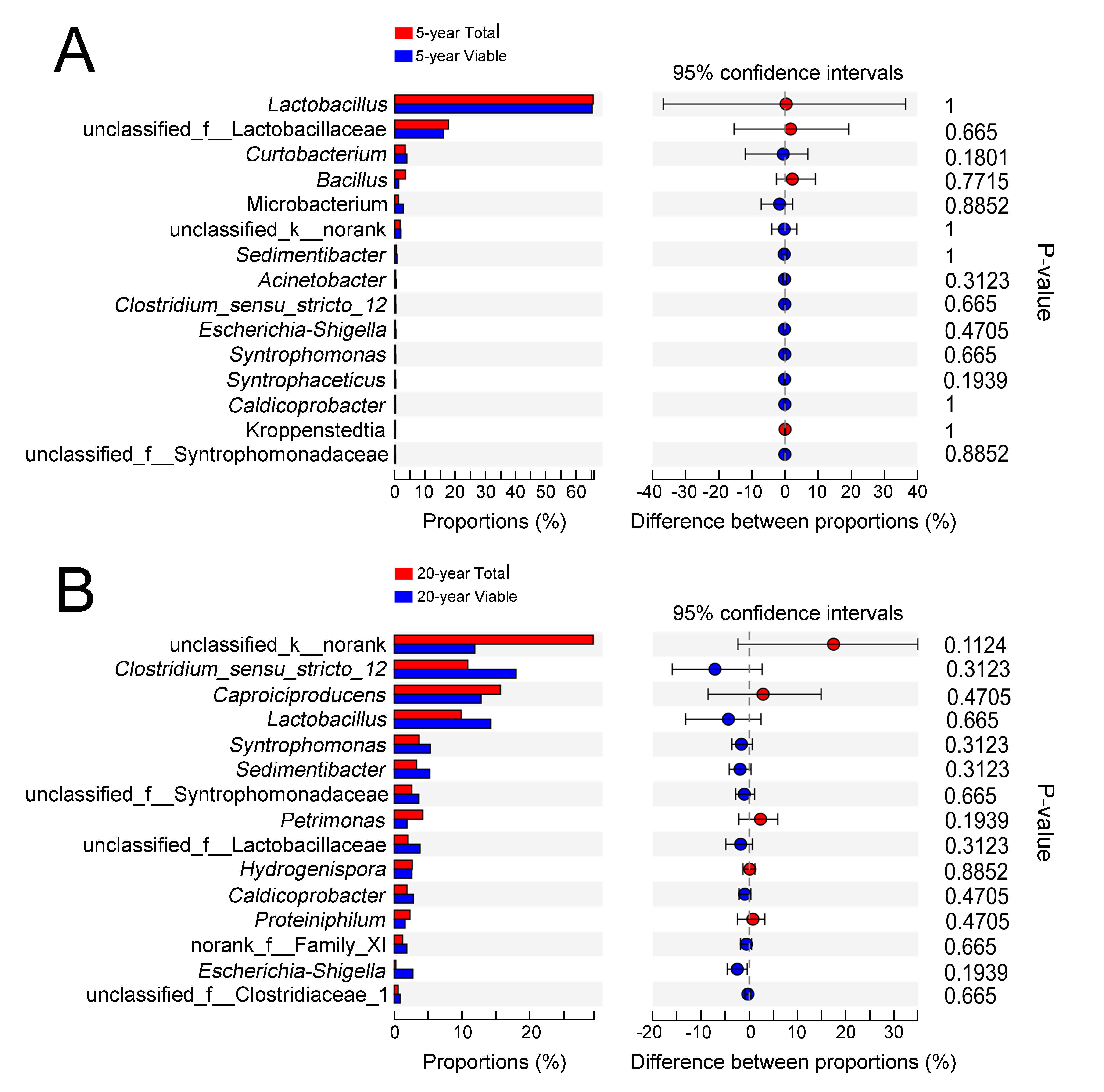


**Supplementary Figure 5. Statistically significant differences in the relative abundance of the top 15 genera between total bacteria and viable bacteria in 5-year PM (A) and 20-year PM (B)**. 5-year PM and 20-year PM were treated with PMA for detecting viable bacteria (5-year Viable, 20-year Viable), and untreated with PMA for detecting total bacteria (5-year Total, 20-year Total).
